# Ältere Menschen in größeren Städten – ein Blick auf die kleinräumige Infrastrukturausstattung

**DOI:** 10.1007/s00391-025-02424-6

**Published:** 2025-03-27

**Authors:** Judith Kaschowitz, Cornelia Müller, Dorothee Winkler

**Affiliations:** Bundesinstitut für Bau‑, Stadt- und Raumforschung (BBSR) im Bundesamt für Bauwesen und Raumordnung (BBR), RS 4 – Städtebauförderung, Soziale Stadtentwicklung, Deichmanns Aue 31–37, 53179 Bonn, Deutschland

**Keywords:** Alterung, Befragungsdaten, Daseinsvorsorge, Sozialräumliche Unterschiede, Geodaten, Ageing, Survey data, Public services, Sociospatial differences, Spatial data

## Abstract

Vor dem Hintergrund der demografischen Alterung in Städten gewinnt die Betrachtung der Lebensbedingungen Älterer an Bedeutung. Mittels kommunalstatistischer Daten der Innerstädtischen Raumbeobachtung und Geodaten wird untersucht, wie viele Einrichtungen der Daseinsvorsorge es in Stadtteilen deutscher Großstädte gibt. Es stehen Stadtteile mit einem hohen Anteil von Personen ab 65 Jahren im Fokus, um Aussagen über die potenzielle Versorgung Älterer mit Infrastruktur zu treffen. Ergänzend dazu werden Befragungsdaten mit kommunalstatistischen Daten verschnitten. Damit kann die Selbsteinschätzung der Wegeentfernungen zu Einrichtungen der Daseinsvorsorge untersucht werden. Im Ergebnis zeigen sich kleinräumige Disparitäten, da signifikante Unterschiede zwischen alten und jungen Stadtteilen (relativ wenige 65-Jährige und Ältere) vorliegen. In alten Stadtteilen gibt es weniger Apotheken und Postfilialen. Die fußläufige Erreichbarkeit von Infrastruktur wird in alten Stadtteilen schlechter eingeschätzt als in jungen Stadtteilen. Insbesondere für hochaltrige und weniger mobile Personen, deren Aktionsradius sich auf das Wohnumfeld beschränkt, kann das problematisch sein. Schlussgefolgert werden kann, dass ein kleinräumiges kommunales Monitoring zentral ist, um Lücken in der Daseinsvorsorge zu erkennen. In weiteren Untersuchungen gilt es, die tatsächliche Nutzung von Infrastrukturen und die Diversität in der Gruppe der Älteren zu berücksichtigen.

## Abstract (en)

Against the background of demographic ageing in cities, it becomes increasingly more important to study the living conditions of old persons. Using municipal statistical data from the Inner City Monitoring System and geodata, the study examined the number of facilities for the provision of services of general interest in the areas of health and the needs of daily living in urban districts of major German cities. The focus is on districts with a high proportion of people aged 65 years and over in order to assess if there is sufficient provision of infrastructure for older people. In addition, survey data are combined with municipal statistical data in order to examine self-assessments of the distances to such facilities. The results show small-scale disparities as there are significant differences between old and young districts (few people aged 65 years and older). There are fewer pharmacies and post offices in older districts. The accessibility of the infrastructure within walking distance is rated worse in old districts than in young districts. This can be particularly problematic for very old and less mobile people whose radius of mobility is limited to the residential area. It can be concluded that small-scale municipal monitoring is crucial to detect gaps in the infrastructure. In further studies, the actual use of infrastructures and the diversity in the group of older people are taken into account.

## Hintergrund und Fragestellung

Auch wenn die Bevölkerung in Städten durchschnittlich jünger ist als in ländlichen Regionen, schreitet die demografische Alterung auch in Städten voran. Eigene Berechnungen auf Basis der Innerstädtischen Raumbeobachtung (IRB, s. Kap. 3) zeigen, dass die Zahl der 65-Jährigen und Älteren in deutschen Großstädten zwischen 2011 und 2022 um 7 % von 4,16 Mio. auf 4,45 Mio. gestiegen ist. Innerhalb von Städten leben ältere Menschen nicht gleich verteilt, sondern überdurchschnittlich häufig am Stadtrand (57 % vs. 51 % der Gesamtbevölkerung). Stadtteile, in denen überproportional viele Ältere leben, zeichnen sich durch eine geringe Bevölkerungsdichte und mehr Wohnfläche je Person aus. Der Anteil von Personen im SGB-II-Bezug (Zweites Buch Sozialgesetzbuch) und mit ausländischer Staatsangehörigkeit ist dort geringer (eig. Berechnungen auf Basis der IRB). Offen ist, welche Infrastrukturen der Daseinsvorsorge in diesen Stadtteilen vorhanden sind, und wie die Erreichbarkeit eingeschätzt wird.

Dieser Beitrag untersucht die Verteilung von Infrastrukturstandorten sowie die Selbsteinschätzung zur Erreichbarkeit in zwei zentralen Bereichen der Daseinsvorsorge: Gesundheit (Arztpraxen, Apotheken) und Bedarfe des täglichen Lebens (Lebensmitteleinzelhandel, Post, Bankautomaten). Der Untersuchungsgegenstand sind alte Stadtteile und Bewohnerinnen und Bewohner in ebendiesen. Kenntnisse über die Verteilung von Infrastrukturstandorten können helfen, Versorgungslücken im Wohnumfeld (operationalisiert über den Stadtteil) zu identifizieren.

## Forschungsstand

Neben individuellen Faktoren (z. B. Gesundheit) und den Wohnverhältnissen (z. B. Zustand der Wohnung) hängt die Selbstständigkeit im Alter auch vom Wohnumfeld (z. B. Nahversorgung) ab [10]. Die Bedeutung des Wohnumfelds wächst mit steigendem Alter bzw. sinkender Mobilität. Personen ab 60 Jahren, aber insbesondere Hochaltrige (80+) verlassen seltener das Haus und legen pro Tag weniger Kilometer zurück als Jüngere [9]. Die Versorgung mit Infrastruktur im Wohnumfeld ist daher insbesondere für mobilitätseingeschränkte Personen relevant, weil das Quartier der zentrale Bezugs- und Handlungsraum ist [1, 2, 4, 11]. In Städten ist die fußläufige Erreichbarkeit dieser Orte relevant, da die Fortbewegung zu Fuß eine hohe Bedeutung hat [9].

Welche Infrastruktur durch Ältere benötigt wird, zeigt eine Übersicht der Weltgesundheitsorganisation (WHO); diese beruht auf qualitativen Interviews, die mit Älteren in 33 internationalen Städten unterschiedlicher Größe geführt wurden. Als für ein selbstständiges Leben wichtig werden Infrastrukturen zum Einkaufen, für Erledigungen und Freizeit genannt. Ältere erwähnen zudem Grünanlagen, Begegnungsorte, Unterstützungsangebote, Freiflächen, Gesundheitsversorgung und Möglichkeiten der sozialen Partizipation [14].

Ob und wie zufrieden Ältere mit der Infrastruktur im Wohnumfeld sind, untersucht eine Studie auf Basis des Deutschen Alterssurvey unter 40- bis 54-, 55- bis 69- und 70- bis 85-Jährigen. Es zeigt sich allgemein eher eine Zufriedenheit mit den Einkaufsmöglichkeiten, wobei die Ältesten zufriedener sind als die Jüngeren, was in der Studie mit sinkenden Ansprüchen Älterer an das Einkaufen erklärt wird. Die medizinische Versorgung wird als ausreichend eingeschätzt. Mobilitätseingeschränkte Personen bewerten z. B. die Einkaufsmöglichkeiten schlechter [10].

Wenige Erkenntnisse liegen jedoch dazu vor, wie die Verteilung von Infrastrukturen der Daseinsvorsorge innerhalb von Städten ist, und wie die Erreichbarkeiten von den Bewohnerinnen und Bewohnern eingeschätzt werden. Die Forschungsfragen lauten: Gibt es Unterschiede in der Verteilung von alltagsrelevanten Infrastrukturstandorten zwischen alten und jungen Stadtteilen in deutschen Großstädten? Wie wird die Erreichbarkeit von Infrastrukturstandorten von den Bewohnerinnen und Bewohnern selbst eingeschätzt?

Aufgrund der Datenverfügbarkeit und zur besseren Vergleichbarkeit mit anderen Studien beschränken wir uns auf die Bereiche Gesundheit und Bedarfe des täglichen Lebens. Der Mehrwert des Beitrags ist, dass auf kleinräumiger Ebene kommunalstatistische und amtliche Daten mit subjektiven Selbsteinschätzungen kombiniert werden und Erkenntnisse zu innerstädtischen räumlichen Disparitäten gewonnen werden.

## Daten und Methode

Datenbasis sind die kommunalstatistischen Daten der IRB, amtliche Geodaten (Points of Interest des Bundes, [3]) und Befragungsdaten des Sozio-oekonomischen Panel (SOEP, bereitgestellt vom DIW Berlin, [13]). Die IRB ist ein Gemeinschaftsprojekt der Kommunalstatistik und des BBSR, an dem sich über 50 deutsche Großstädte beteiligen. Die Städte stellen jährlich über 400 Merkmale auf Stadtteilebene bereit [5]. Weiter wird die IRB-Altersklassifikation, die Stadtteile, über alle Städte hinweg, anhand der Quartile der Altersverteilung den Kategorien jung, gemischt und alt zuordnet, genutzt. Alte Stadtteile sind diejenigen mit einem hohen Anteil älterer Bevölkerung (65-Jährige und Ältere) und einem niedrigen oder mittleren Anteil junger Bevölkerung (18- bis 29-Jährige), während junge Stadtteile hohe Anteile 18- bis unter 29-Jähriger und niedrige oder mittlere Anteile älterer Bevölkerung aufweisen. Alle übrigen Stadtteile fallen in die Kategorie gemischt [6]. Auch die Lage der Stadtteile wird in den Analysen berücksichtigt, da sich Infrastrukturstandorte meist nicht gleichmäßig über die Stadt verteilen. Dazu werden Stadteile gemäß ihrer räumlichen Lage der Innenstadt, dem Innenstadtrand oder Stadtrand zugeordnet [5]. Es können 2609 Stadtteile in 53 Städten analysiert werden (Tab. 1).

**Tab. 1:** Anzahl der Stadtteile nach Altersklassifikation

Altersklassifikation	
Jung	542
Gemischt	1544
Alt	523
Gesamt	2609

Daten: IRB des BBSR 2022 (Kommunalstatistiken der IRB-Städte); eig. Berechnung; *n* = 53 Städte

Die ausgewählten Infrastrukturstandorte (Points of Interest, POI) stammen vom Bundesamt für Kartographie und Geodäsie (BKG) [3]. Genutzt werden Daten zu Arztpraxen, Apotheken, Lebensmitteleinzelhandel, Postfilialen und Bankautomaten. Diese werden mit den IRB-Daten auf Stadtteilebene verschnitten. Jedes Infrastrukturmerkmal wird als durchschnittliche Anzahl der jeweiligen Infrastruktur pro 1000 Einwohnerinnen und Einwohner (EW) im Stadtteil berechnet. Nach einer deskriptiven Auswertung der Merkmale nach der Altersklassifikation führen wir lineare Regressionsanalysen durch; bei diesen wird das jeweilige Infrastrukturmerkmal als metrische abhängige Variable, die in Zusammenhang mit der Altersklassifikation gebracht wird (unabhängige Variable), verwendet, wobei die Lage des Stadtteils berücksichtigt wird (Kontrollvariable). Aussagen, die auf Basis dieser Regressionsanalysen gemacht werden können, sind z. B., dass es in alten vs. jungen Stadtteilen eine niedrigere/höhere Anzahl eines bestimmten Infrastrukturmerkmals gibt.

Um eine subjektive Einschätzung zu erhalten, wird das SOEP mit den IRB-Daten verschnitten und deskriptiv ausgewertet. Der Datensatz enthält somit Befragte, die in den Stadtteilen der 53 Städten leben. Aus dem SOEP wird die fußläufige Erreichbarkeit („Wie lange brauchen Sie, um zu Fuß die folgenden Einrichtungen in Ihrem Wohngebiet zu erreichen?“) zu Hausarzt/-ärztin, Bankautomaten und Geschäften des täglichen Bedarfs in zwei Altersgruppen (unter 65 Jahre und 65+) für Befragte, die in alten, jungen oder gemischten Stadtteilen leben, analysiert. Mit dem Fokus auf diese Infrastrukturmerkmale kann die Vergleichbarkeit zu den Ergebnissen auf Basis der IRB und Geodaten hergestellt werden. Die Altersgruppen können aufgrund der zu kleinen Fallzahlen nicht weiter ausdifferenziert werden.

## Ergebnisse

**Abb. 1:**
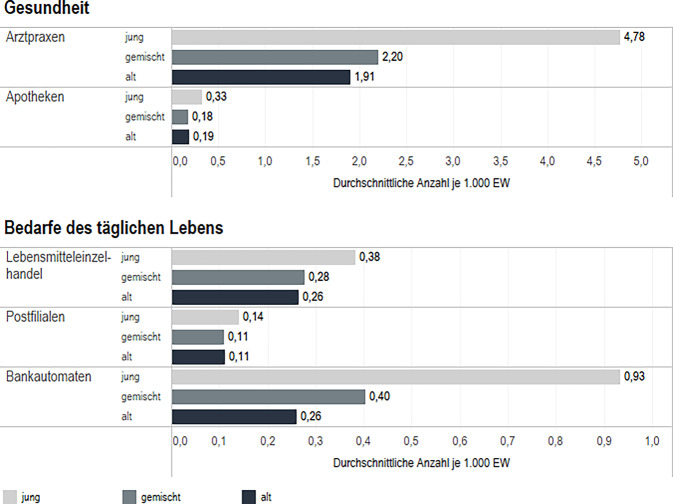
Infrastrukturmerkmale in Stadtteilen nach Altersklassifikation. Daten: IRB des BBSR 2022 (Kommunalstatistiken der IRB-Städte); BKG (2023); eig. Berechnung*/*Darstellung.; *n* = 53 Städte, 2568 Stadtteile (jung *n* = 538; gemischt *n* = 1518, alt *n* = 512)

Es gibt Unterschiede zwischen den Stadtteilen in Bezug auf die Verteilung der Infrastrukturstandorte je 1000 EW. In alten Stadtteilen ist die relative Zahl der Arztpraxen und Apotheken etwa halb so hoch wie in jungen Stadteilen. Es gibt, relativ betrachtet, weniger Lebensmitteleinzelhandel und Bankautomaten in alten im Vergleich zu jungen Stadtteilen. Kaum Unterschiede bestehen bei Postfilialen (Abb. 1). Eine mögliche Erklärung dafür ist die Lage alter Stadtteile am Stadtrand.

Um möglichst unverzerrte Ergebnisse zu erhalten, wird in den multivariaten Analysen geprüft, ob sich der festgestellte Zusammenhang zwischen der Altersklassifikation und der Ausstattung mit Infrastruktur noch zeigt, wenn der Einfluss der Lage auf den Zusammenhang herausgerechnet wird.

Die Regressionsergebnisse zeigen, dass es, unter Kontrolle der Lage, in alten Stadtteilen signifikant weniger Apotheken (0,066/1000 EW) als in jungen Stadtteilen gibt, aber kein Unterschied bei der Anzahl der Arztpraxen besteht (Tab. 2). Weiter gibt es in alten Stadtteilen signifikant weniger Postfilialen (0,084/1000 EW) als in jungen Stadtteilen, aber es besteht kein Unterschied zwischen den Stadtteilen bei der Verteilung der Lebensmitteleinzelhandel und Bankautomaten.

**Tab. 2:** Regressionsergebnisse Infrastrukturmerkmale

	Arztpraxen	Apotheken	Lebensmitteleinzelhandel	Postfilialen	Bankautomaten
Altersklassifikation	Regressionskoeffizient (Standardfehler)				
Alt	−0,520 (0,618)	−0,066*** (0,021)	−0,132 (0,084)	−0,084*** (0,022)	−0,059 (0,186)
Gemischt	−0,669 (0,504)	−0,084*** (0,017)	−0,053 (0,068)	−0,078*** (0,018)	−0,211 (0,152)
Jung (Ref.)	–	–	–	–	–
	*n* = 2609	*n* = 2609	*n* = 2609	*n* = 2609	*n* = 2609

Daten: IRB des BBSR 2022 (Kommunalstatistiken der IRB-Städte); BKG (2023); eig. Berechnung; Kontrollvariable: Lage (Innenstadt, Innenstadtrand, Stadtrand)

Signifikanzniveau: *p < 0,05, **p < 0,01, ***p < 0,001

**Abb. 2:**
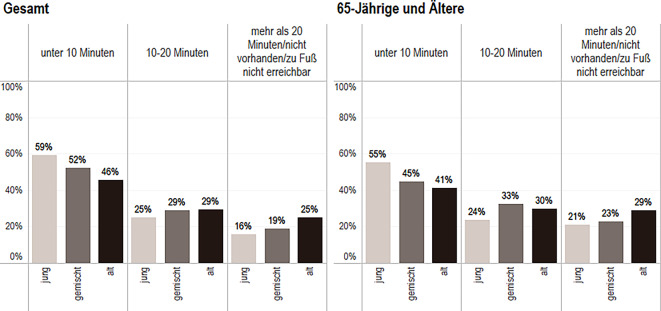
Einschätzung der fußläufigen Erreichbarkeit des Hausarztes/der Hausärztin. Daten: IRB des BBSR 2022 (Kommunalstatistiken der IRB-Städte); SOEP 2019 (v38); eig. Berechnung und Darstellung.; *n* = 4985 Personen (*Gesamt*: jung *n* = 1216, gemischt *n* = 2985, alt *n* = 784; *65-Jährige und Ältere*: jung *n* = 218, gemischt *n* = 642, alt *n* = 255)

Die Verschneidung der SOEP- und IRB-Daten ermöglicht eine Betrachtung der Einschätzung zur fußläufigen Erreichbarkeit (Abb. 2, 3 und 4). Leider liegen zu Apotheken und Postfilialen keine Informationen vor. Befragte in alten Stadtteilen geben zu 46 % an, ihren/ihre Hausarzt/-ärztin in unter 10 min erreichen zu können und liegen damit 14 Prozentpunkte unter der Einschätzung Befragter in jungen Stadtteilen. Weiter zeigt sich, dass Ältere (65 Jahre und älter) in alten Stadtteilen die Erreichbarkeit von Hausarzt/-ärztin mit etwa 40 % um etwa 5 Prozentpunkte schlechter einschätzen als alle Bewohnerinnen und Bewohner alter Stadtteile.

**Abb. 3:**
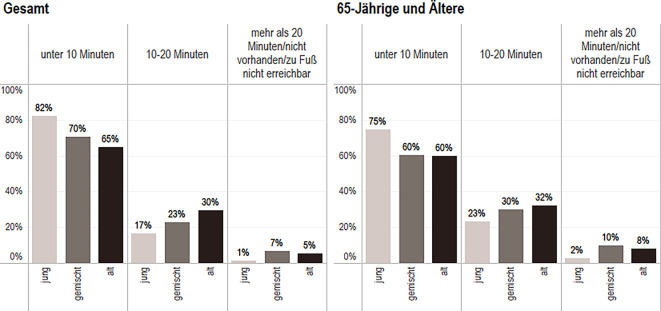
Einschätzung der fußläufigen Erreichbarkeit der Geschäfte des täglichen Bedarfs. Daten: IRB des BBSR 2022 (Kommunalstatistiken der IRB-Städte); SOEP 2019 (v38); eig. Berechnung und Darstellung; *n* = 5078 Personen (*Gesamt*: jung *n* = 1237, gemischt *n* = 3043, alt *n* = 798; *65-Jährige und Ältere*: jung *n* = 222, gemischt *n* = 652, alt *n* = 257)

**Abb. 4:**
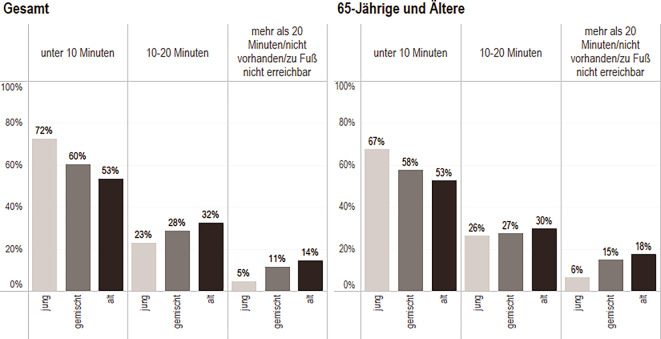
Einschätzung der fußläufigen Erreichbarkeit eines Bankautomaten. Daten: IRB des BBSR 2022 (Kommunalstatistiken der IRB-Städte); SOEP 2019 (v38); eig. Berechnung und Darstellung.; *n* = 5069 Personen (*Gesamt*: jung *n* = 1234; gemischt *n* = 3040, alt *n* = 795; *65-Jährige und Ältere*: jung *n* = 219, gemischt *n* = 650, alt *n* = 255)

Geschäfte des täglichen Bedarfs können laut Selbsteinschätzung überwiegend in unter 10 min erreicht werden. Dennoch bestehen die Unterschiede nach der Alterszusammensetzung im Stadtteil und Zugehörigkeit zu einer Altersgruppe weiter. Insgesamt geben Befragte in alten Stadtteilen zu 65 % an, Geschäfte des täglichen Bedarfs in unter 10 min erreichen zu können, im Gegensatz zu 82 % in jungen Stadtteilen. Für ältere Befragte (65+) in alten Stadtteilen stellt sich zudem die Erreichbarkeit mit ca. 60 % um 5 Prozentpunkte schlechter dar als die Gesamtangabe.

Die Zustimmungsraten zur Erreichbarkeit von Bankautomaten liegen zwischen der Zustimmung zu Erreichbarkeit von Geschäften des täglichen Bedarfs und zur Erreichbarkeit von Hausarzt/-ärztin. Die Unterschiede in der Zustimmung nach der Alterszusammensetzung im Stadtteil (jung, gemischt, alt) bestehen ebenfalls. Etwa die Hälfte der Befragten in alten Stadtteilen (53 %) gibt an, einen Bankautomaten in unter 10 min erreichen zu können vs. 73 % in jungen Stadtteilen. Die Angaben der 65-Jährigen und Älteren unterscheiden sich jedoch mit 53 % nicht vom Gesamtwert.

## Diskussion

Ziel der Analyse war es, Aussagen über die Daseinsvorsorge in Stadtteilen zu treffen, in denen der Anteil Älterer hoch ist. Dazu wurden ausgewählte Infrastrukturmerkmale betrachtet. Die Ergebnisse zeigen, dass alte Stadtteile, relativ betrachtet, über weniger wohnortnahe Infrastruktur verfügen als junge Stadtteile. Es gibt signifikant weniger Apotheken und Postfilialen. Da im SOEP die Erreichbarkeit von Apotheken und Postfilialen nicht abgefragt wird, konnten diese beiden Einrichtungen im zweiten Analyseteil nicht berücksichtigt werden. Grundsätzlich schätzen alle Bewohnerinnen und Bewohner in alten Stadtteilen die Erreichbarkeit von Infrastruktur schlechter ein als alle Bewohnerinnen und Bewohner in jungen Stadteilen. Innerhalb der Stadtteile gibt es zudem Unterschiede nach Alter. Befragte, die 65 Jahre oder älter sind, schätzen die fußläufige Erreichbarkeit überwiegend schlechter ein als alle Bewohnerinnen und Bewohner des Stadtteils. Die Abweichungen liegen jedoch maximal bei 5 Prozentpunkten. Die deskriptiven und multivariaten Ergebnisse zeigen, dass gemischte Stadtteile alten Stadtteile ähneln und sich fast gleichermaßen von jungen Stadtteilen, z. B. in der Infrastrukturausstattung, unterscheiden. Diesen Unterschieden sollte in weiteren Untersuchungen nachgegangen werden.

Die Ergebnisse zeigen Unterschiede in der Infrastrukturausstattung für die Bereiche Gesundheit sowie Geschäfte des täglichen Bedarfs auf und offenbaren innerstädtische Disparitäten. Daraus resultieren unterschiedliche Infrastrukturangebote für ältere Menschen innerhalb von Städten, die sich auf deren Handlungsmöglichkeiten auswirken. Da die Versorgung im Wohnumfeld insbesondere für Ältere wichtig ist, kann die Selbstständigkeit wenig mobiler Älterer in betroffenen Stadtteilen eingeschränkt sein.

Die Ergebnisse erweitern den Forschungsstand zur subjektiven Bewertung des Wohnumfelds und zur objektiven Betrachtung der innerstädtischen Daseinsvorsorge, da hier die Infrastrukturausstattung in Abhängigkeit vom Alter der Bewohnerschaft sowie die individuelle Bewertung der Erreichbarkeit der Infrastruktur in Abhängigkeit vom Alter auf Stadtteilebene betrachtet werden. Methodisch hervorzuheben sind die kleinräumige Analyse verschiedener Datenarten und die Kombination objektiver und subjektiver Daten zur Daseinsvorsorge. Der Abgleich zeigt, dass sich die subjektive Wahrnehmung mit dem objektiven Vorhandensein von Infrastruktur grundsätzlich deckt, auch wenn das in dieser Analyse nicht für einzelne Stadtteile verglichen werden kann. Es können keine Rückschlüsse auf einzelne Städte oder bestimmte Stadtteile erfolgen, da eine stadtübergreifende Analyse erfolgte.

## Limitationen

Diese Einschränkungen gelten:

- Verwendet wird die Anzahl an Infrastrukturen vor Ort, d. h. der tatsächliche Zugang; die Nutzung und Qualität der Angebote bleiben unberücksichtigt.
- Nicht alle Älteren sind von ihrem Wohnumfeld abhängig; das könnte in zukünftigen Analysen und mit besserer Datenverfügbarkeit durch eine Analyse weiterer Altersgruppen berücksichtigt werden.
- Die Analyse nutzt administrative Grenzen zur Annäherung an die Wohnumgebung.

## Ausblick

Das Alter(n) in der Stadt zu berücksichtigen, bleibt eine wichtige Aufgabe der Stadtentwicklung. Die Infrastrukturplanung ist eine kommunale Querschnittsaufgabe, bei der verschiedene Stellen wie die Stadt‑, Alten- und Verkehrsplanung eng zusammenarbeiten müssen. Ein regelmäßiges Monitoring zum Vorhandensein zentraler Infrastruktur hilft, Handlungsbedarfe zu erkennen. Damit Ältere im gewohnten Wohnumfeld bleiben können, gilt es, die wohnortnahe Daseinsvorsorge, d. h. die räumliche Verteilung der Infrastrukturstandorte, zu beobachten, um ggf. nachsteuern zu können. Daneben ist die Beseitigung von Barrieren wichtig, um allen Bevölkerungsgruppen Zugang zu ermöglichen. Zu beachten ist, dass durch die zunehmende Digitalisierung neue Barrieren entstehen können. Eine gezielte Quartiersentwicklung, in der Planende die „veränderte[n] Bedürfnisse älter werdender Menschen differenziert aufzeigen und darauf eine Antwort geben …“ [15], kann das Altern im gewohnten Wohnumfeld unterstützen. Ein Ansatz ist die Zusammenlegung von z. B. Arztpraxis, Bank- und Postfiliale an einem gut erreichbaren Standort – auch wenn manche Firmenstrategie, z. B. die der Deutschen Post, die zurzeit einen Abbau wohnortnaher Postfilialen diskutiert, dem entgegensteht. Auch in (Bundes‑)Förderprogrammen sollten die Bedarfe Älterer mitgedacht werden. In Zeiten begrenzter Haushaltsmittel ist allerdings zu berücksichtigen, dass Leistungen der öffentlichen Hand nicht allen Älteren (kostenlos) zur Verfügung gestellt werden können, sondern sich an Bedarfen orientieren sollten. Vor dem Hinblick der weiteren Alterung in den Städten in den nächsten Jahren [8], ist auch der Blick auf zukünftige Ältere wichtig. Eine Studie zeigte, dass momentan 55- bis 64- und 65- bis 74-Jährige mit dem Infrastrukturangebot im Wohnumfeld im Hinblick auf das eigene Älterwerden nicht zufrieden sind, d. h., dieses als nicht ausreichend empfinden [12].

Innerhalb von Städten bestehen Unterschiede in der wohnortnahen Infrastrukturversorgung, die quartiersspezifisch beobachtet und auch im Hinblick auf die Diversität und Bedarfe im Alter bewertet werden müssen. Neben der Verfügbarkeit von Infrastruktur ist der Abbau von Barrieren, insbesondere für Personen mit geringen Ressourcen, für die Nutzung wichtig. Die Zusammenarbeit planender Stellen in Kommunen ist zentral.

Künftige Untersuchungen zum Alter(n) in Städten sollten interdisziplinäre Bezüge herstellen und die Diversität im Alter stärker berücksichtigen. Auch wenn ein Teil der Älteren bis ins hohe Alter aktiv und unabhängig ist und von den Vorzügen des städtischen Lebens profitiert, wird ein anderer Teil, weniger gesund und mobil, stärker von der direkten wohnortnahen Versorgung abhängig sein [12]. Zudem unterscheiden sich die individuellen finanziellen Ressourcen. Nicht für alle Älteren ist es wichtig, Infrastruktur im Wohnumfeld zu haben, da z. B. Lieferdienste genutzt werden, die Mobilität mit dem eigenen Pkw garantiert ist und Unterstützung durch in der Nähe lebende Verwandte oder Nachbarinnen und Nachbarn erfolgt. Gleichzeitig ist zu berücksichtigen, dass sich die Lebenswelten der nachkommenden Älteren von denen der jetzigen Älteren unterscheiden und sich Bedarfe möglicherweise ändern [7].

## Data Availability

Die hier verwendeten Daten können nach dem Abschluss von Nutzungsvereinbarungen genutzt werden. Für das SOEP erfolgt die Beantragung über das DIW (https://www.diw.de/de/diw_01.c.601584.de/datenzugang.html). Zur Nutzung der IRB-Daten ist das Postfach stadtbeobachtung@bbr.bund.de zu kontaktieren.
